# Future global annual frozen ground distribution datasets based on Frost Number Model with Kappa coefficient

**DOI:** 10.1038/s41597-026-06918-9

**Published:** 2026-02-28

**Authors:** Xiaoduo Pan, Hu Li, Xiaowei Nie

**Affiliations:** 1 National Tibetan Plateau Data Center, State Key Laboratory of Tibetan Plateau Earth System, Environment and Resources (TPESER), Institute of Tibetan Plateau Research, Chinese Academy of Sciences, Beijing, 100101 China; 2 University of the Chinese Academy of Sciences, Beijing, 100049 China; 3 School of Economics and Management, University of the Chinese Academy of Sciences, Beijing, 100049 China; 4 School of Ecology and Environment, Xizang University, Lhasa, 850000 China

**Keywords:** Cryospheric science, Projection and prediction

## Abstract

Permafrost, which underlies roughly one-fifth of global land area, is highly vulnerable to ongoing climate warming and its degradation has important implications for hydrological processes, carbon cycling, ecosystems, and infrastructure stability. It remains challenging to accurately project its future distribution due to various factors. The Surface Frost Index (SFI) threshold is a critical parameter that captures first-order thermal forcing for projecting future permafrost distribution. Here, the future global annual frozen ground distribution datasets are produced using an optimized frost number threshold (*F*_*at*_) calibrated via kappa coefficient-based accuracy assessment. By comparing simulated outputs with benchmark maps, we identify the optimal *F*_*at*_. Applying this threshold to downscaled CMIP6 data (0.25° resolution), we generate annual frozen ground distributions (2020–2099) under four SSP scenarios (SSP126, SSP245, SSP370, SSP585). The datasets reveal accelerated permafrost degradation, with mid-century (2040–2060) losses of 19 ± 3% to 28 ± 3% and late-century (2080–2099) losses escalating to 21 ± 3% to 61 ± 5%, peaking under SSP585. These high-resolution projections provide critical insights for assessing climate impacts and guiding cryospheric adaptation strategies.

## Background & Summary

Frozen ground, an important component of the cryosphere, is composed of soil or rock containing ice and encompasses permafrost, seasonally frozen ground, and intermittently frozen ground^1,2^. Permafrost is defined as the ground remains frozen for two or more consecutive years, covering approximately one quarter of the land area in the Northern Hemisphere^3^. Seasonally frozen ground represents regions where the near-surface soil freezes from 15 days up to but not including two consecutive years, while intermittently frozen ground refers to regions where the soil is frozen for less than 15 days per year^4^. Changes in frozen ground have important impacts on regional water cycles^5,6^, carbon cycles^7,8^, ecological environment^9,10^, and climate system feedbacks^11,12^.

Permafrost is widely distributed in high-latitude regions of the Northern Hemisphere such as Siberia, Alaska, and northern Canada, as well as in high-altitude regions such as the Qinghai-Xizang Plateau, the Alps, and the Andes^13–17^. The first map of permafrost in the Northern Hemisphere, the *Circum-Arctic Map of Permafrost and Ground Ice Conditions*^18,19^, was produced by the International Permafrost Association (IPA) in the 1990s by integrating all available data and regional permafrost maps. According to the *IPA permafrost map*, the permafrost region covers approximately 22.79 × 10^6^ km^2^ in the Northern Hemisphere^4^, while the estimated area actually underlain by permafrost ranges from 12.21 × 10^6^ km^2^ to 16.98 × 10^6^ km^2^ ^20,21^. In addition, Obu^22^ clarified that because each permafrost zone (continuous, discontinuous, sporadic, and isolated) is not entirely underlain by permafrost, the actual permafrost area is approximately 14 × 10^6^ km^2^, accounting for about 15% of the exposed land surface area in the Northern Hemisphere. The maximum range of seasonally frozen ground is about 48.12 × 10^6^ km^2^, accounting for 50.5% of the land area of the Northern Hemisphere, and the area of intermittently frozen ground is about 6.27 × 10^6^ km^2^, accounting for 6.6% of the land area of the Northern Hemisphere^4^.

Under the background of global warming, permafrost is projected to degrade further in the future^23,24^. At present, the models used to simulate the distribution of frozen ground include the Response Model^25^, the Mean Annual Ground Temperature Model^26^, the Altitude Model^27^, and the Frost Number Model^28^. In addition, some Earth System Models (ESMs) can also be used for permafrost projections^29,30^. Among these approaches, the Frost Number Model is still widely used. In previous studies^31,32^, the permafrost distribution is diagnosed with a certain threshold (*F*_*gt*_ = 0.5) based on the surface frost number calculated from the ground freezing/thawing index. Although CMIP6 models provide both ground temperature and air temperature as standard output variables, air temperature from the NEX-GDDP-CMIP6 datasets^33^ is selected because the datasets apply daily variant of the monthly bias correction/spatial disaggregation (BCSD) method^34^ to CMIP6 data, which reducing systematic errors in CMIP6 data and providing enhanced spatial resolution (0.25°). By utilizing air temperature from the NEX-GDDP-CMIP6 datasets, the accuracy and spatial resolution of the simulation of permafrost distribution can be improved compared to directly using the CMIP6 output. Therefore, the diagnosing results heavily depend on the threshold (*F*_*at*_), which is not certain and varies across different studies, and the method for accurately determining this threshold remains unclear. Guo and Wang^35^ used the air temperature from the Coupled Model Intercomparison Project Phase 5 (CMIP5) in the frost number model with *F*_*at*_ = 0.60 to diagnose the permafrost. Chang, *et al*.^36^ calculated the *F*_*a*_ value with *F*_*at*_ = 0.58 as the threshold to diagnose the permafrost in the Qinghai-Xizang Plateau.

Here, we present a novel method to get the threshold (*F*_*at*_) to diagnose the global historical and future distributions of frozen ground based on the frost number model with Kappa coefficient, and then simulate the global future distribution of frozen ground under SSP126, SSP245, SSP370, and SSP585 scenarios.

## Methods

### Data

#### Existing permafrost distribution maps

The *high-resolution permafrost distribution maps in the Northern Hemisphere (2000–2016)*^37,38^ are produced by integrating large amounts of field data and multisource geospatial data, especially remote sensing data with the method of statistical learning model, which is more accurate than previous circumpolar maps. This map is served as the benchmark map for diagnosing the optimal frost number threshold for the distribution of permafrost in this study.

Ran, *et al*.^39^ integrated multiple existing permafrost maps with simulated permafrost distribution map for the Tibetan Plateau, unifying data acquisition times across different regions of China to produce a comprehensive frozen ground map of China^39,40^. This map divides China’s frozen ground into high-latitude permafrost, high-altitude permafrost, plateau permafrost, alpine permafrost, medium seasonal frozen ground, shallow seasonal frozen ground, intermittently frozen ground, and unfrozen ground. These categories are reclassified as follows: permafrost (high-latitude permafrost, high-altitude permafrost, alpine permafrost and plateau permafrost), seasonally frozen ground (medium seasonal frozen ground and shallow seasonal frozen ground), intermittently frozen ground, and unfrozen ground. This map serves as the benchmark map to diagnose the optimal frost number thresholds for the distribution of seasonally frozen ground and intermittently frozen ground.

The *IPA permafrost distribution map*^18,19^ represents the distribution of permafrost in the Northern Hemisphere before 1990. According to the continuity and underground ice content, the permafrost is divided into 20 categories. It is used to verify the simulated permafrost distribution in the Northern Hemisphere before 1990.

The *permafrost map of Russia (1996)*^41^ represents the permafrost extent of Russia, which was digitized as ESRI Shapefiles from paper maps (scale 1:20,000,000 to 1:40,000,000)^42^, with polygons assigned attributes based on the classes used in the legends of the paper map. This map serves for the validation of simulated permafrost distribution map in Russia.

The *Permafrost, Atlas of Canada, 5th Edition (1978–1995)*^43^ shows the extent of permafrost in Canada during the period from 1978 to 1995, which is published by Natural Resources Canada. This map is utilized for validating the simulated permafrost distribution map in Canada.

#### NEX-GDDP-CMIP6 datasets

The air temperature from the NEX-GDDP-CMIP6 datasets^33^ is used to calculate the air freezing/thawing index to simulate the distribution of frozen ground. The NEX-GDDP-CMIP6 datasets are downscaled by the bias correction/seasonal disaggregation (BCSD) method^34^ based on CMIP6. The horizontal resolution of the datasets is 0.25°. Multi-model datasets from five scenarios (historical, SSP126, SSP245, SSP370 and SSP585) are used in this study.

#### Topographic data

GTOPO30^44^ is a global DEM with a horizontal grid spacing of 1 km, which is produced based on multi-source data, including vector and raster data. In this study, the GTOPO30 data is resampled to 0.25° to ensure consistency with the horizontal resolution of the simulated permafrost distribution map. This data is employed to diagnose regions where high-altitude permafrost may exist.

**Method.** Based on the air frost number model^45^, the optimal *F*_*at*_ is obtained based on the air frost number model and the Kappa coefficient. The equation of the air frost number model is:

1 $${F}_{a}=\frac{\sqrt{{{DDF}}_{a}}}{\sqrt{{{DDF}}_{a}}+\sqrt{{{DDT}}_{a}}}$$

The air freezing index ${{DDF}}_{a}$ is the sum of the daily average air temperature below 0 °C, which can be calculated as:

2 $$DD{F}_{{\rm{a}}}=\mathop{\sum }\limits_{i}^{N}|{T}_{i}|dt,{T}_{i} < 0^\circ {\rm{C}}$$

where ${T}_{i}$ is the daily mean air temperature, and the freezing period consists of days $i=\mathrm{1,2},\ldots ,N$. The freezing period is defined from July to June of the following year to capture the freezing index during a continuous cold season, ensuring that the entire winter freezing period is included within a single calculation cycle^46,47^.

The air thawing index ${{DDT}}_{a}$ is the sum of the daily average air temperature above 0 °C, which can be calculated as:

3 $$DD{T}_{{\rm{a}}}=\mathop{\sum }\limits_{i}^{M}|{T}_{i}|dt,{T}_{i} > {0}^{\circ }{\rm{C}}$$

where the thawing period consists of days $i=\mathrm{1,2},\ldots ,M$. The thawing process typically occurs within a single calendar year in frozen ground regions. Therefore, the thawing period refers to the continuous warm season from January to December of the year^46,47^.

The key to the novel method is to accurately find the optimal *F*_*at*_ to determine the distribution of frozen ground. Here, a novel method to determine the threshold (*F*_*at*_) for diagnosing the global historical and future distributions of frozen ground based on the frost number model with Kappa coefficient, which has been evaluated over the Qinghai-Xizang Plateau and proved to be a valid method^48^. Firstly, values of *F*_*at*_ which range from 0 to 1, with an interval of 0.01, are assigned to diagnose the distribution of frozen ground. Then, the simulated frozen ground distribution maps are compared with the existing frozen ground distribution map. The classification accuracy of the simulated frozen ground maps is evaluated using the Kappa coefficient^49,50^. Finally, the optimal *F*_*at*_ with the highest Kappa coefficient can be determined to accurately determine the distribution of frozen ground. Kappa coefficients of >0.8, 0.61–0.8, 0.41–0.6, 0.21–0.4, and 0–0.2 indicate almost perfect, substantial, moderate, fair, and low degrees of fit, respectively, between the two maps^51^. The equations are:

4 $${p}_{e}=\frac{{{\rm{a}}}_{1}\times {{\rm{b}}}_{1}+{{\rm{a}}}_{2}\times {{\rm{b}}}_{2}+\ldots +{{\rm{a}}}_{c}\times {{\rm{b}}}_{c}}{{\rm{n}}\times {\rm{n}}}$$

where ${p}_{e}$ represents the overall proportion of chance-expected agreement. ${{\rm{a}}}_{1},{{\rm{a}}}_{2},\ldots ,{{\rm{a}}}_{c}$ are the numbers of actual samples for each category, ${{\rm{b}}}_{1},{{\rm{b}}}_{2},\ldots ,{{\rm{b}}}_{c}$ are the numbers of predicted samples for each category, and ${\rm{n}}$ is the total number of samples.

5 $${p}_{o}=\frac{{{\rm{b}}}_{11}+{{\rm{b}}}_{22}+\ldots +{{\rm{b}}}_{{cc}}}{{\rm{n}}}$$

where ${p}_{o}$ represents the overall accuracy. ${{\rm{b}}}_{11},{{\rm{b}}}_{22},\ldots ,{{\rm{b}}}_{{cc}}$ are the number of samples correctly classified in each category.

Then the kappa coefficient is calculated as follows:

6 $$k=\frac{{p}_{o}-{p}_{e}}{1-{p}_{e}}$$

**Fig. 1:**
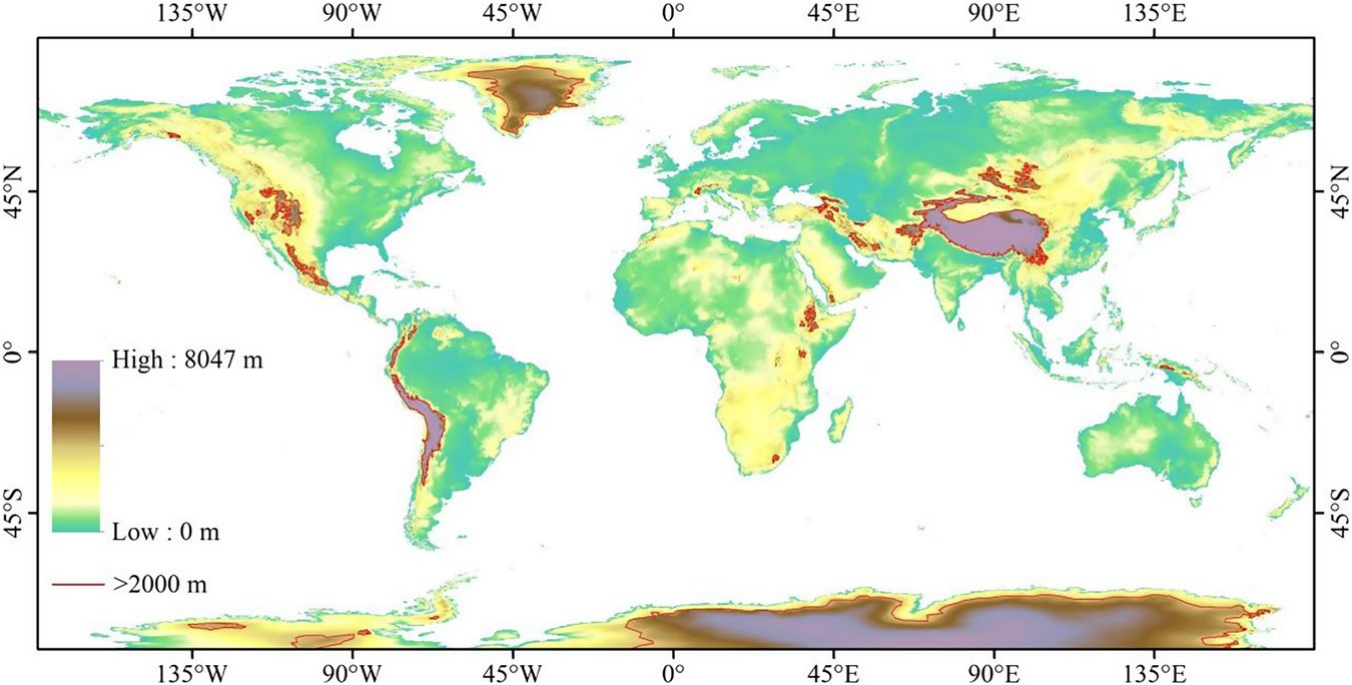
Global map of elevation. The areas enclosed by the red solid line indicate elevations higher than 2000 meters.

Globally, permafrost is widely distributed in high-latitude regions, as well as in high mountain regions and high-altitude plateaus in mid- and low-latitude regions^13,52^. The distribution of high-latitude permafrost is mainly affected by latitude, while the distribution of high-altitude permafrost is influenced by both altitude and latitude^13,53^. Therefore, the relationship between the distribution of high-altitude permafrost regions and air temperature differs from that of high-latitude permafrost regions and air temperature. Based on the GTOPO30 global elevation map^44^, the regions with altitudes above 2000 m are identified as potential high-altitude permafrost regions^54^, mainly located in the TP, the Cordillera Mountains, Antarctica, and Greenland (Fig. 1). High-latitude is defined as areas located above 60° latitude in both the Northern and Southern Hemispheres^55^. The high-latitude permafrost region and high-altitude permafrost region were distinguished to determine the optimal *F*_*at1*_ for determining the permafrost region, where *F*_*a*_ ≥ *F*_*at1*_ represents the permafrost region.

**Fig. 2:**
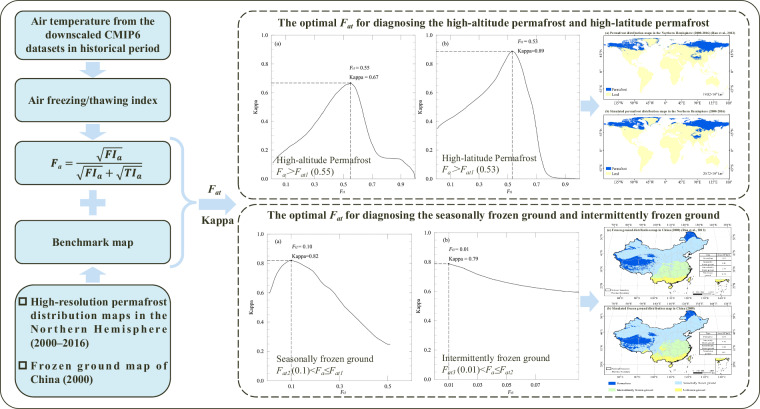
Flowchart illustrating the novel method for simulating the global distribution of frozen ground.

The flowchart of the novel method for diagnosing frozen ground distribution is shown in Fig. 2. Compared with the *High-resolution permafrost distribution maps in the Northern Hemisphere (2000–2016)*, the Kappa coefficient is used as the measure of classification accuracy to obtain the optimal *F*_*at*_. Considering the difference between the high-latitude permafrost region and the high-altitude permafrost region, *F*_*at1*_ = 0.55 (Kappa = 0.67) is obtained as the optimal frost number threshold for diagnosing the high-altitude permafrost region, and *F*_*at1*_ = 0.53 (Kappa = 0.89) is obtained as the optimal frost number threshold for diagnosing the high-latitude permafrost region.

The optimal frost number thresholds *F*_*at2*_ and *F*_*at3*_ for diagnosing the distribution of seasonally frozen ground and intermittently frozen ground are further obtained. There are few internationally published global distribution maps of seasonally frozen ground and intermittently frozen ground. Therefore, to simulate the global distribution of seasonally frozen ground and intermittently frozen ground, the *Frozen ground map of China (2000)*^39,40^ is used as the benchmark map, which classifies frozen ground into three categories: permafrost, seasonally frozen ground, and intermittently frozen ground. Then, the optimal frost number thresholds for diagnosing seasonally frozen ground (*F*_*at2*_ = 0.10) and intermittently frozen ground (*F*_*at3*_ = 0.01) are obtained to simulate the global frozen ground. *F*_*at2*_ ≤ *F*_*a*_ < *F*_*at1*_ represents the seasonally frozen ground, and *F*_*at3*_ ≤ *F*_*a*_ < *F*_*at2*_ represents the intermittently frozen ground.

## Data Records

The global frozen ground distribution datasets (1950–2099) generated in this study have been publicly released and can be downloaded from the National Tibetan Plateau Data Center (TPDC), and the link is 10.11888/Cryos.tpdc.300901^56^. The “frozen” variable in the datasets uses integer values to classify frozen ground types: 0 represents unfrozen ground, 1 represents intermittently frozen ground, 2 represents seasonally frozen ground, and 3 represents permafrost. The datasets are provided in NetCDF format and can be used with NCAR Command Language (NCL), Python, and ArcGIS software. TPDC provides comprehensive metadata including data summary, keywords, thumbnails, spatiotemporal resolution, data coverage, and citation information^57^. Detailed information can be accessed through the TPDC website.

The datasets are stored based on the namelist of CMIP6 models used in this study (Table 1). Within each model folder, frozen ground distribution data for five different scenarios are organized into subdirectories: Historical, SSP126, SSP245, SSP370, and SSP585. The files follow a standardized naming convention: ModelName_Frozen_xxxx_yyyy.nc, where “xxxx” represents the scenarios and “yyyy” indicates the year. All datasets are provided in NetCDF format, ensuring compatibility with widely-used analysis tools including NCL, Python, and ArcGIS software.

**Table 1:** Namelist of CMIP6 Models output in this study.

GCM Name	Modeling center	References
ACCESS-CM2	Commonwealth Scientific and Industrial Research Organisation (CSIRO)	Bi, *et al*.^58^
ACCESS-ESM1-5	Commonwealth Scientific and Industrial Research Organisation (CSIRO)	Ziehn, *et al*.^59^
BCC-CSM2-MR	Beijing Climate Center, China	Xin, *et al*.^60^
CanESM5	Canadian Centre for Climate Modelling and Analysis (CCCma)	Swart, *et al*.^61^
CMCC-ESM2	Euro-Mediterranean Centre	Lovato, *et al*.^62^
CNRM-CM6-1	Centre National de Recherches Météorologiques (CNRM) and Cerfacs	Voldoire, *et al*.^63^
CNRM-ESM2-1	Centre National de Recherches Météorologiques (CNRM) and Cerfacs	Séférian, *et al*.^64^
EC-Earth3	EC-Earth consortium	Döscher, *et al*.^65^
EC-Earth3-Veg-LR	EC-Earth consortium	Döscher, *et al*.^65^
FGOALS-g3	Institute of Atmospheric Physics Chinese Academy of Sciences	Li, *et al*.^66^
GFDL-ESM4	Geophysical Fluid Dynamics Laboratory (GFDL)	Horowitz, *et al*.^67^
GISS-E2-1-G	NASA Goddard Institute for Space Studies	Kelley, *et al*.^68^
INM-CM4-8	Institute for Numerical Mathematics (INM)	Volodin^69^
INM-CM5-0	Institute for Numerical Mathematics (INM)	Volodin^70^
IPSL-CM6A-LR	Institute Pierre-Simon Laplace Climate Modeling Center (IPSL CMC)	Boucher, *et al*.^71^
KACE-1-0-G	National Institute for Materials Science (NIMS)	Halder, *et al*.^72^
MIROC-ES2L	Japanese climate modeling group	Hajima, *et al*.^73^
MIROC6	Japanese climate modeling group	Tatebe, *et al*.^74^
MPI-ESM1-2-HR	Max-Planck-Institute for Meteorology	Müller, *et al*.^75^
MPI-ESM1-2-LR	Max-Planck-Institute for Meteorology	Mauritsen, *et al*.^76^
MRI-ESM2-0	Meteorological Research Institute	Adachi, *et al*.^77^
NorESM2-LM	Norwegian Climate Center	Seland, *et al*.^78^
NorESM2-MM	Norwegian Climate Center	Seland, *et al*.^78^
TaiESM1	Research Center for Environmental Changes	Wang, *et al*.^79^
UKESM1-0-LL	Met Office Hadley Centre (MOHC)	Sellar, *et al*.^80^

## Technical Validation

Based on the optimal frost number thresholds and historical CMIP6 temperature data, maps of historical global frozen ground distribution were simulated to conduct robust validation. The *IPA permafrost distribution map*^18,19^, the *permafrost map of Russia (1996)*^41^, and the *Permafrost, Atlas of Canada, 5th Edition (1978–1995)*^43^ are used to evaluated the simulated frozen ground distribution datasets.

**Fig. 3:**
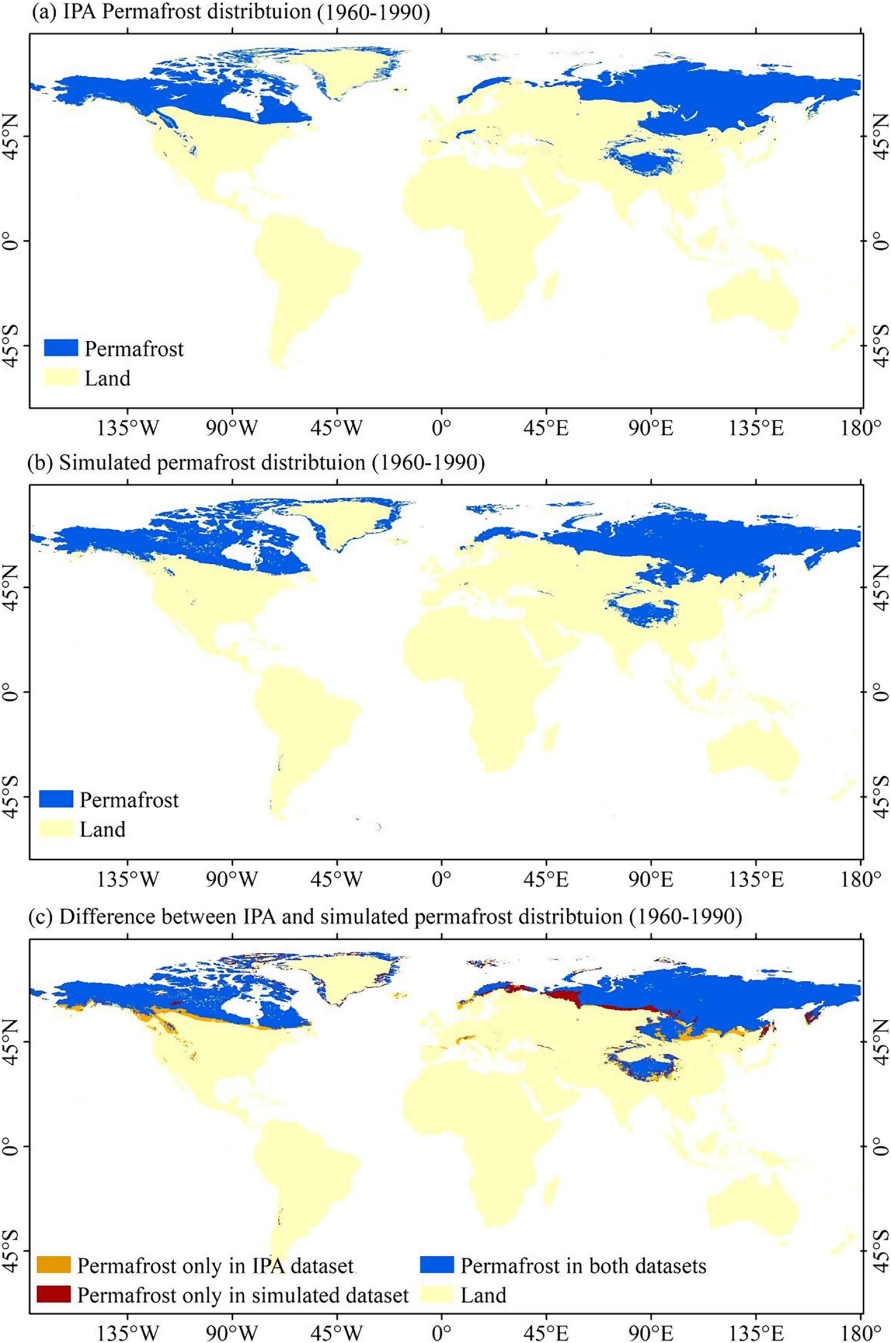
(**a**) IPA map of the permafrost distribution map; (**b**) simulated permafrost map from 1960 to 1990 based on CMIP6 multi-model ensemble mean; (**c**) difference between IPA and simulated permafrost regions (1960–1990).

Globally, Fig. 3 shows the comparison of the *IPA permafrost map*^18,19^ with the simulated permafrost maps from 1960 to 1990. To generate the simulated permafrost map, the annual freezing index (*DDF*_*a*_) and thawing index (*DDT*_*a*_) for each year from 1960 to 1990 were first calculated using daily air temperature data. The multi-year mean freezing and thawing indices over this 30-year period were then computed to derive the climatological frost number, which was used to diagnose permafrost distribution based on the optimal frost number threshold. The simulated area of permafrost region is 23.72 × 10^6^ km^2^, which is close to the area of IPA permafrost region at 22.79 × 10^6^ km^2^. The areal bias of the permafrost region is 0.93 × 10^6^ km^2^. The Kappa coefficient is 0.76, indicating that the simulated permafrost map is highly consistent with the *IPA permafrost map*.

**Fig. 4:**
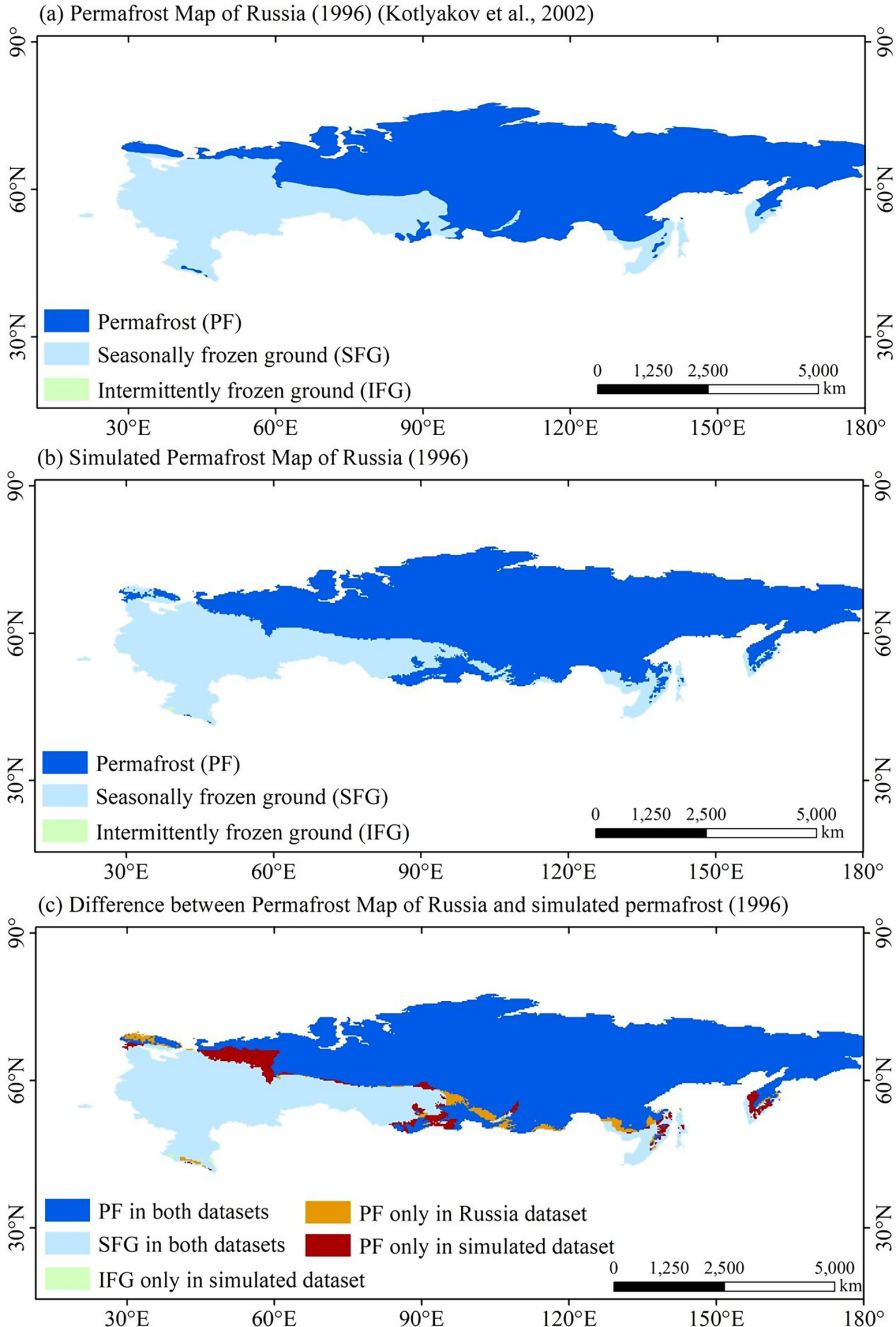
(**a**) Permafrost map for Russia (1996); (**b**) simulated permafrost map for Russia (1996) based on CMIP6 multi-model ensemble mean; (**c**) difference between Permafrost Map of Russia and simulated permafrost (1996).

Figure 4 shows that the simulated permafrost distribution map is highly consistent with the *Permafrost map of Russia*^41^. The simulated map was generated for the year 1996 using CMIP6 daily air temperature data to match the temporal coverage of the observational dataset (1 January 1996 to 31 December 1996). The annual freezing index (*DDF*_*a*_) and thawing index (*DDT*_*a*_) for 1996 were calculated, from which the frost number was derived and used to diagnose permafrost distribution based on the optimal frost number threshold. The Kappa coefficient between the two maps is 0.87, demonstrating excellent spatial agreement between the simulated permafrost map and the *Permafrost map of Russia*. The area of permafrost region in the *Permafrost map of Russia*^41^ and the simulated permafrost map of Russia is 10.72 × 10^6^ km^2^ and 11.01 × 10^6 ^km^2^, respectively. The areal bias is only 0.29 × 10^6^ km^2^, indicating that the simulated permafrost map accurately reproduces the permafrost distribution in Russia.

**Fig. 5:**
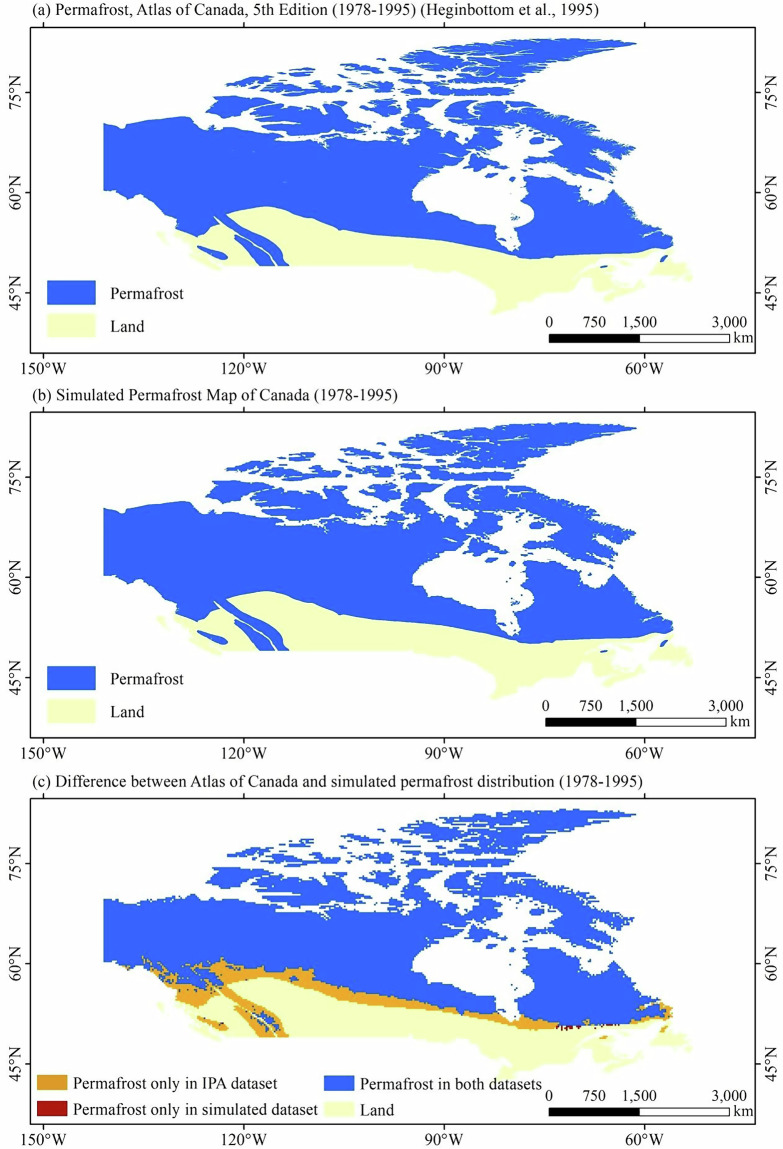
(**a**) Permafrost, Atlas of Canada, 5th Edition (1978–1995); (**b**) the simulated permafrost distribution map of Canada (1978–1995) based on CMIP6 multi-model ensemble mean; (**c**) difference between Atlas of Canada and simulated permafrost distribution map.

Over Canada, compared with the *Permafrost, Atlas of Canada, 5th Edition (1978–1995)*^43^, the simulated permafrost map of Canada accurately reproduces the distribution of permafrost region in Canada (Fig. 5). The simulated permafrost map of Canada was generated using the climatological frost number derived from multi-year mean (1978–1995) freezing and thawing indices calculated from CMIP6 daily air temperature data, matching the temporal coverage of the Atlas of Canada. The area of permafrost region in the *Permafrost, Atlas of Canada, 5th Edition (1978–1995)*^43^ and the simulated permafrost map of Canada are 7.10 × 10^6 ^km^2^ and 6.08 × 10^6 ^km^2^, respectively. The Kappa coefficient between the two maps is 0.78, indicating that the two maps are substantial agreement.

**Fig. 6:**
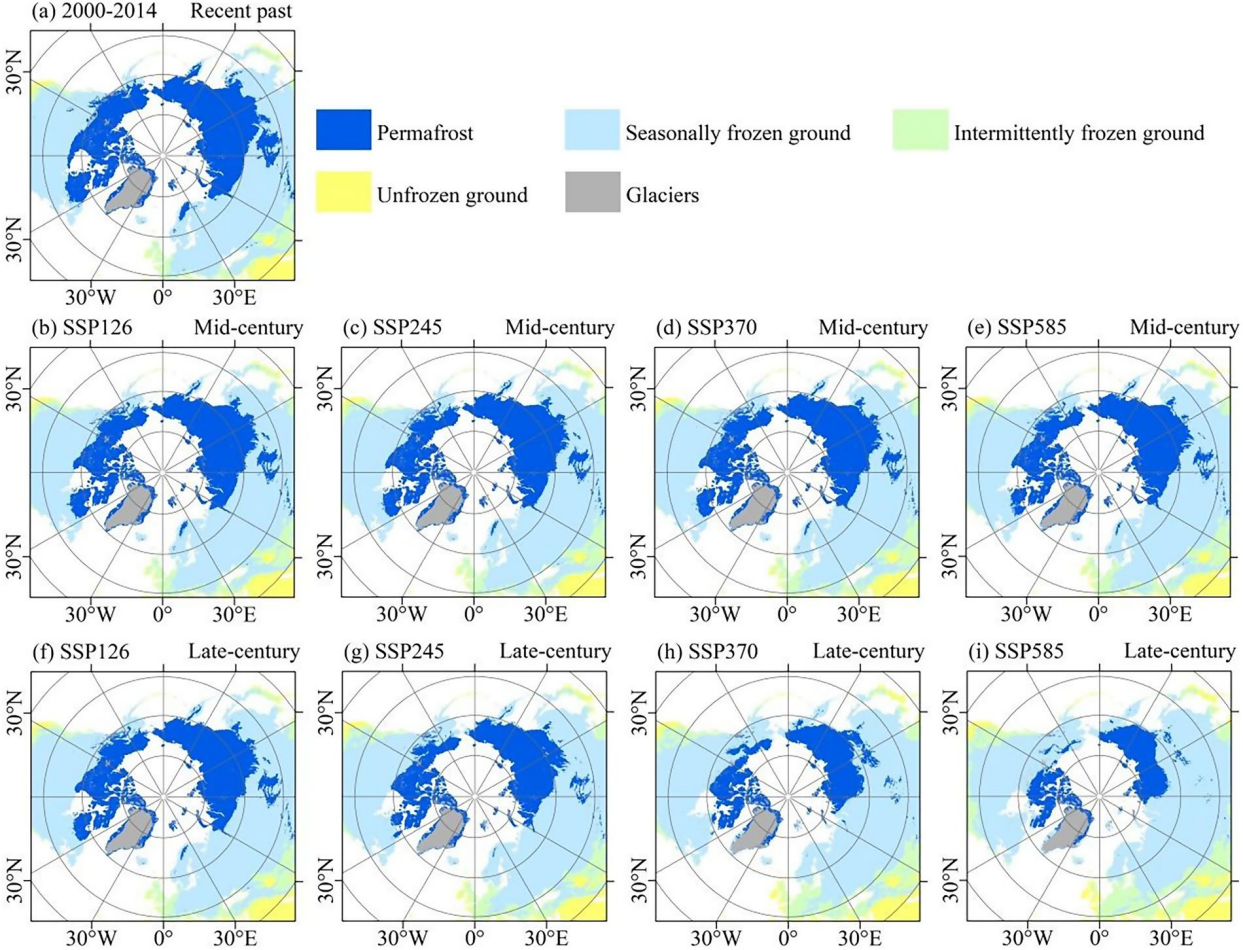
Frozen ground distribution in the Arctic under different scenarios and time periods based on CMIP6 multi-model ensemble mean. The glacier distribution shown in the figure is derived from the Randolph Glacier Inventory (RGI)^81^. (**a**) represents the recent past (2000–2014); (**b**), (**c**), (**d**), and (**e**) represent the mid-century (2040–2060) under the SSP126, SSP245, SSP370, and SSP585 scenarios, respectively; (**f**), (**g**), (**h**), and (**i**) represent the late-century (2080–2099) under the SSP126, SSP245, SSP370, and SSP585 scenarios, respectively.

The spatial distribution patterns of global frozen ground under different scenarios and periods are analyzed. Figure 6 shows the frozen ground distribution under different scenarios and periods. During the mid-century (2040–2060), the spatial distribution patterns of the permafrost region remain similar under different scenarios. The degradation of the permafrost region is primarily concentrated at the southern edge of the continuous permafrost, gradually retreating towards higher latitude areas. During the late-century (2080–2099), the spatial distribution patterns of permafrost exhibit significant changes under different scenarios. In the SSP126 and SSP245 scenarios, the spatial patterns of the permafrost region are similar between the mid and late-century, suggesting relatively stable conditions. However, under the SSP370 and SSP585 scenarios, the spatial distribution of the permafrost region undergoes substantial changes, both in the mid and late-century. Especially in the late-century, the high-altitude permafrost in the Qinghai-Xizang Plateau has almost completely degraded, and the high-latitude permafrost has also been degraded significantly. These findings highlight the vulnerability of permafrost to climate change, with the permafrost region being particularly susceptible to degradation, especially in high-altitude regions.

**Fig. 7:**
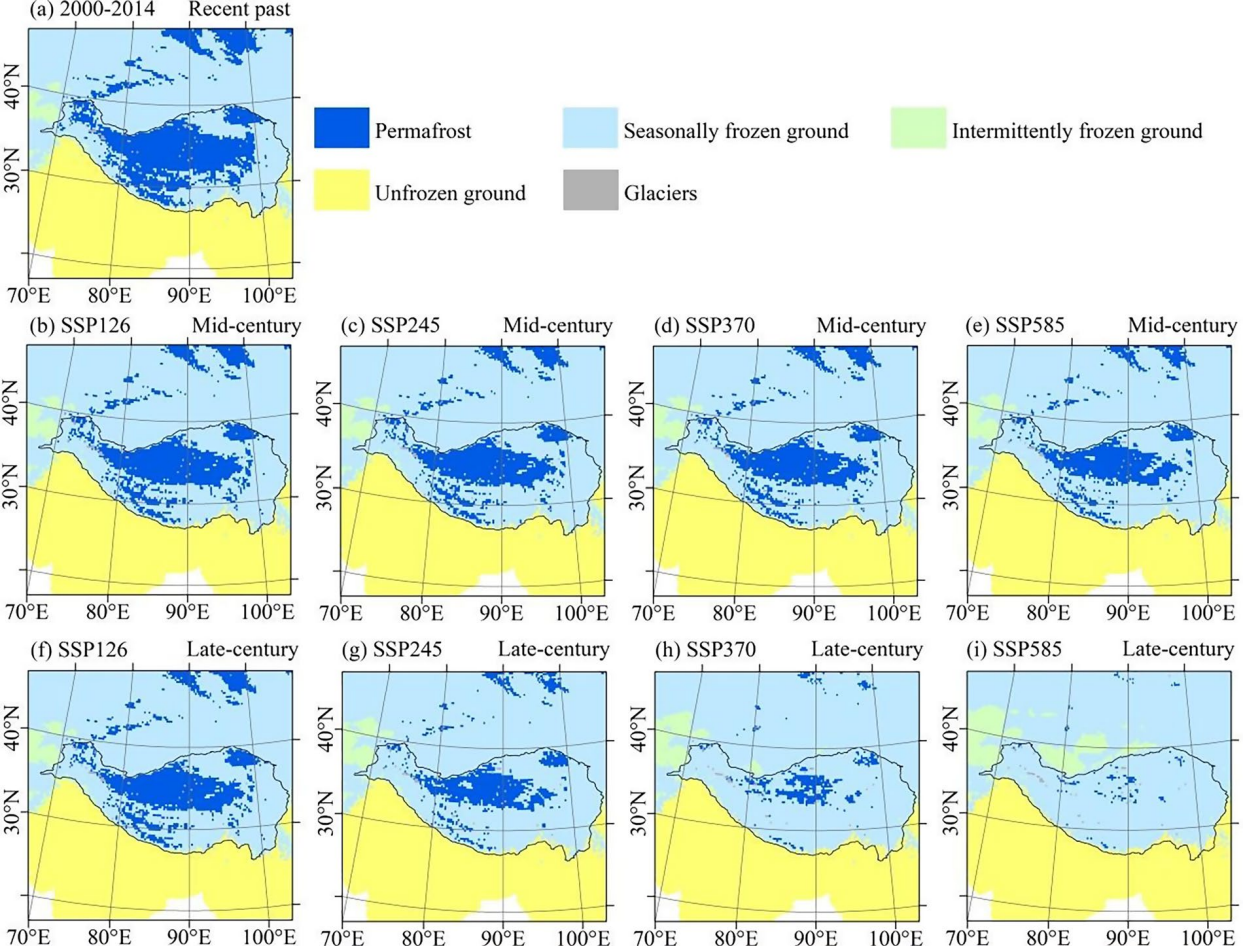
Frozen ground distribution in the Third Pole under different scenarios and time periods based on CMIP6 multi-model ensemble mean. The glacier distribution shown in the figure is derived from the Randolph Glacier Inventory (RGI)^81^. (**a**) represents the recent past (2000–2014); (**b**), (**c**), (**d**), and (**e**) represent the mid-century (2040–2060) under the SSP126, SSP245, SSP370, and SSP585 scenarios, respectively; (**f**), (**g**), (**h**), and (**i**) represent the late-century (2080–2099) under the SSP126, SSP245, SSP370, and SSP585 scenarios, respectively.

Under the context of global warming, frozen ground distribution is projected to undergo significant changes across the Arctic (Fig. 6) and Third Pole regions (Fig. 7). In the Arctic, where the most extensive permafrost coverage is observed globally, permafrost is projected to degrade from its southern edges, transitioning to seasonally frozen ground and intermittently frozen ground. In Antarctica, which is predominantly characterized by ice sheets, intermittently frozen ground is primarily distributed in coastal ice-free areas. Under future climate scenarios, only slight changes are projected in the area and distribution of intermittently frozen ground in Antarctic regions. In the Third Pole region, permafrost is projected to undergo substantial degrade. Particularly under the SSP585 scenario, permafrost in the Third Pole is projected to be almost entirely transformed into seasonally frozen ground in the late-century.

Globally, permafrost is projected to degrade significantly, while seasonally frozen ground and intermittently frozen ground are projected to increase. Table 2 shows the changes in the global frozen ground area under different scenarios and periods. Compared with the permafrost region in the recent past (2000–2014), the permafrost region has degraded by 19 ± 3% and 21 ± 3% under the SSP126 scenario, indicating that the degradation rate of permafrost region has gradually decreased from the recent past to the late-century. The seasonally frozen ground and intermittently frozen ground show an increasing trend. During the mid-century and late-century, the seasonally frozen ground increases by 5 ± 1% and 6 ± 1%, respectively, and the intermittently frozen ground increases by 9 ± 2% and 10 ± 2%, respectively. Under the SSP245 scenario, the area of permafrost region degrades by 22 ± 3% and 35 ± 4% in the mid-century and late-century, respectively. Both seasonally frozen ground and intermittently frozen ground show a significant increasing trend in the future. Under the SSP370 scenario, the degradation rate of the permafrost region is close to that under the SSP245 scenario in the mid-century, with 25 ± 3% of permafrost degradation. During the late-century, the degradation rate of permafrost will accelerate. By the end of the 21^st^ century, 52 ± 4% of the global permafrost region will be degraded. Under the SSP585 scenario, 28 ± 3% of permafrost region will be degraded in the mid-century. In the late-century, the degradation rate of permafrost region is projected to be faster, with permafrost degradation rate of 61 ± 5%. The seasonally frozen ground and intermittently frozen ground are projected to increase by 11 ± 1% and 37 ± 5%, respectively.

**Table 2:** Rate of change (multi-model mean ± 90% confidence interval) of the global frozen ground for different scenarios and periods.

	SSP126		SSP245		SSP370		SSP585	
	Mid century	Late century	Mid century	Late century	Mid century	Late century	Mid century	Late century
Permafrost region	−19 ± 3%	−21 ± 3%	−22 ± 3%	−35 ± 4%	−25 ± 3%	−52 ± 4%	−28 ± 3%	−61 ± 5%
Seasonally frozen ground	5 ± 1%	6 ± 1%	6 ± 1%	8 ± 1%	6 ± 1%	11 ± 1%	7 ± 1%	11 ± 1%
Intermittently frozen ground	9 ± 2%	10 ± 2%	10 ± 2%	17 ± 3%	12 ± 3%	28 ± 4%	15 ± 3%	37 ± 5%

**Fig. 8:**
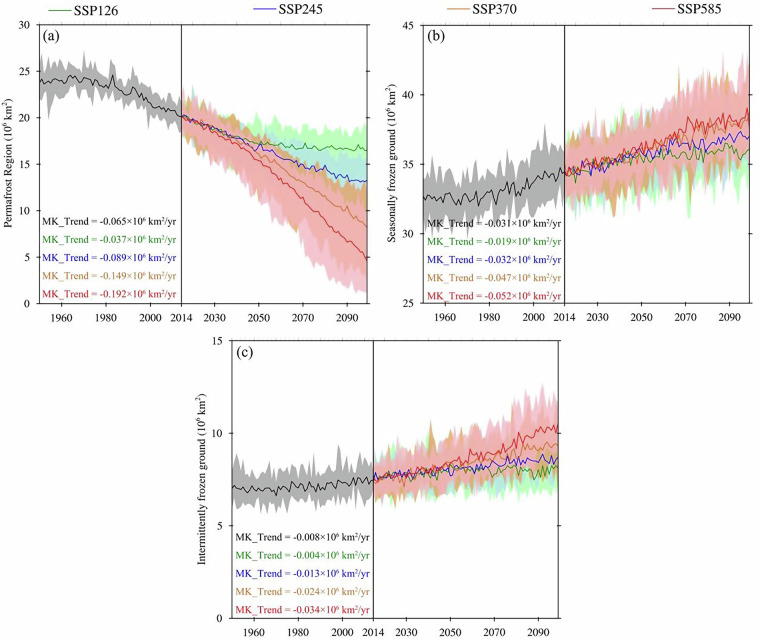
Projected changes in (**a**) permafrost, (**b**) seasonally frozen ground, and (**c**) intermittently frozen ground during period from 1950 to 2099. The top and bottom bounds of the shaded region are the maximum and minimum of the area from the CMIP6 models.

The area of permafrost shows a decreasing trend under different scenarios, especially under the SSP585 scenario. Figure 8(a) shows projected changes in permafrost region during period from 1950 to 2099. From 1950 to 2099, the area of permafrost region shows a significant decreasing trend. During the historical period (1950–2014), the area of permafrost decreases at a rate of 0.065 × 10^6^ km^2^/yr. Under the SSP126 scenario, characterized by sustainable development and robust climate change mitigation measures, the degradation rate of the permafrost region is relatively lower than that observed during the historical period, declining at a rate of 0.037 × 10^6 ^km^2^/yr. This finding underscores the efficacy of proactive strategies aimed at curbing greenhouse gas emissions and mitigating the loss of permafrost. Under the SSP245 scenario, representing a moderate greenhouse gas emissions trajectory, the degradation rate of the permafrost region is close to that observed in the historical period, indicating that in the absence of substantial efforts to mitigate climate change and adapt to its impacts, the permafrost region will continue to experience substantial degradation at a rate of 0.089 × 10^6 ^km^2^/yr. Under the SSP370 scenario, characterized by high greenhouse gas emissions and limited climate change mitigation, the permafrost region undergoes an accelerated degradation rate, with a rate of 0.149 × 10^6 ^km^2^/yr. The SSP585 scenario, reflecting a future characterized by high greenhouse gas emissions and limited adaptation measures, exhibits the most rapid degradation rate among all scenarios. The permafrost region under this scenario is projected to degrade at a rate of 0.192 × 10^6^ km^2^/yr.

The area of seasonally frozen ground and intermittently frozen ground shows an increasing trend under different scenarios. Figure 8(b,c) show the changing trends of seasonally frozen ground and intermittently frozen ground from 1950 to 2099, providing valuable insights into their changing dynamics under different periods and scenarios. The seasonally frozen ground and intermittently frozen ground show an increasing trend under different periods and scenarios. For seasonally frozen ground, the area increases at a rate of 0.031 × 10^6 ^km^2^/yr in the historical period. Under the SSP126, SSP245, SSP370 and SSP585 scenarios, the area of seasonally frozen ground increases at rates of 0.019 × 10^6 ^km^2^/yr, 0.032 × 10^6 ^km^2^/yr, 0.047 × 10^6 ^km^2^/yr and 0.052 × 10^6 ^km^2^/yr, respectively. Under the SSP245 scenario, the change trend of seasonally frozen ground is close to that in the historical period. The intermittently frozen ground increases at a rate of 0.008 × 10^6 ^km^2^/yr in the historical period. Under the SSP126, SSP245, SSP370 and SSP585 scenarios, the area of intermittently frozen ground increases at rates of 0.004 × 10^6 ^km^2^/yr, 0.013 × 10^6 ^km^2^/yr, 0.024 × 10^6 ^km^2^/yr and 0.034 × 10^6 ^km^2^/yr, respectively.

## Usage Notes

The datasets provide global frozen ground distribution maps with annual temporal resolution at 0.25° spatial resolution, covering the period from 1950 to 2099. The data values represent different types of frozen ground: “3” represents permafrost, “2” represents seasonally frozen ground, “1” represents intermittently frozen ground, and “0” represents unfrozen ground. The datasets provide valuable data support for understanding changes in frozen ground and their response to climate change, enabling better assessment of impacts on hydrological and carbon cycles, ecological environments, and engineering construction in Cryospheric regions. Additionally, the publicly available datasets can support climate change impact assessments and adaptation planning in vulnerable regions affected by permafrost degradation.

## Data Availability

The datasets are accessible without restrictions in the National Tibetan Plateau Data Center (TPDC) under the following link: 10.11888/Cryos.tpdc.300901^56^. The datasets are provided in NetCDF format and can be used with NCL, Python, and ArcGIS software.

## Code availability

The code used for generating the future global frozen ground distribution maps was developed using NCL and Python. Both versions can be found at https://github.com/TPDC-HL/Code-for-global-frozen-ground-distribution-maps-1950-2099.
